# Development and validation of the TexCoMP model: A nonlinear framework for optimising slip-resistant walkway coatings in diverse environments

**DOI:** 10.1371/journal.pone.0350565

**Published:** 2026-06-15

**Authors:** In-Ju Kim

**Affiliations:** Department of Industrial Engineering and Engineering Management, College of Engineering, University of Sharjah, Sharjah, United Arab Emirates; IGDTUW: Indira Gandhi Delhi Technical University for Women, INDIA

## Abstract

**Background:**

Slip-related accidents remain a significant safety concern in public walkways, healthcare facilities, and industrial environments, particularly under contaminated surface conditions. Although numerous studies have investigated slip resistance using friction measurements and statistical classification approaches, existing predictive models often rely on simplified linear relationships or data-driven algorithms that provide limited physical interpretability of tribological interactions.

**Objectives:**

This study aims to experimentally evaluate the slip-resistance performance of coated ceramic walkway surfaces under varying environmental conditions and to develop an interpretable, nonlinear, predictive framework, termed TexCoMP (Texture-Coating-Material-Performance), that integrates coating properties, surface texture characteristics, and environmental contamination effects. Specific objectives are to: (1) compare four coatings across four ceramic tiles and three shoe types; (2) assess arid, damp, and foamy environmental impacts; and (3) identify optimal combinations for walkway safety per ANSI A137.1:2022 and ISO 5436:2021 standards.

**Methods:**

Four coating materials (CM 1-CM 4) were applied to four ceramic tile surfaces (CT 1-CT 4) and evaluated using tribological testing under arid, damp, and foamy contamination conditions with three footwear materials. Surface texture parameters were characterised using roughness measurements, and dynamic friction coefficients (DFCs) were determined through controlled dynamic friction tests. A nonlinear predictive model incorporating coating material performance (CMP), surface texture modification (STM), and environmental factors (E) was developed and validated using statistical analysis and cross-validation across 144 experimental combinations (432 individual measurements).

**Results:**

TexCoMP achieved a mean absolute error (MAE) of 0.03, a root mean square error (RMSE) of 0.04, a Pearson correlation (*R*) of 0.92, and an *R*^2^ of 0.93, outperforming linear models by 22% in terms of RMSE under foamy conditions. The epoxy-CT4 pairing yielded a DFC of 0.62 ± 0.03 in damp conditions, 24% above the OSHA safety threshold of 0.5, indicating substantially reduced slip potential. Four-way ANOVA revealed highly significant interactions (*p* < 0.001) with environmental condition as the dominant factor (partial η² = 0.47).

**Conclusions:**

The proposed TexCoMP framework provides an interpretable tribological model for predicting slip resistance in coated walkway systems by integrating coating material properties, surface texture modification, and environmental contamination effects. The results demonstrate that coatings incorporating texture-enhancing particles can significantly improve friction performance under damp conditions. TexCoMP therefore offers a practical analytical tool for guiding coating selection and surface engineering strategies to reduce slip-related risks in built environments.

## Introduction

Slip-related falls represent a pressing public and workplace safety and health concern, contributing to significant physical injuries and economic losses worldwide [1]. The National Floor Safety Institute reports that falls account for over 8 million hospital emergency room visits annually in the United States alone, making them the leading cause of such visits [2]. Similarly, the Centers for Disease Control and Prevention (CDC) estimates that fall-related injuries cost billions of dollars yearly in medical expenses and lost productivity, with workplace incidents exacerbating these figures through compensation claims and absenteeism [3]. These statistics highlight the importance of enhancing slip resistance in flooring surfaces, a crucial factor in mitigating fall risks across residential, commercial, and industrial environments.

Slip resistance is typically quantified using the dynamic coefficient of friction (DFC) between footwear and flooring surfaces, which reflects the tribological interaction at the shoe-floor interface. This interaction is influenced by multiple factors, including surface texture characteristics, footwear materials, coating properties, and environmental conditions [4–6]. Maintaining sufficient friction is particularly important in environments where contamination by water, detergents, or other fluids may reduce traction and increase slip risk. To improve surface performance under such conditions, various floor coatings—such as epoxy, polyurethane, acrylic, and polymer-based systems—are commonly applied to flooring substrates, including concrete, wood, and ceramic tiles [7]. These coatings can enhance durability, surface roughness, and aesthetic qualities while also modifying tribological behaviour. For example, acid-based etchants can increase micro-scale surface roughness to improve mechanical interlocking, epoxy coatings can create textured and wear-resistant surfaces, and polyurethane coatings provide flexibility and resilience under varying environmental conditions [8–11]. Nevertheless, the effectiveness of these coating systems often varies depending on the combined influence of surface texture characteristics and environmental contamination conditions, and these interactions remain insufficiently understood.

Prior studies have advanced slip-resistance research but have revealed critical gaps in scope and integration. Khaday et al. measured friction on stone and ceramic floors under arid, damp, and foamy conditions. However, they focused solely on inherent surface roughness, without exploring coating strategies, limiting their applicability to treated surfaces [12]. Similarly, Smith and Lee demonstrated that coatings with enhanced hardness and elasticity improve friction but failed to develop a predictive model for texture-environment interactions, limiting applicability [13]. Blanco et al. investigated nanoparticle-enhanced epoxy coatings, demonstrating improved slip resistance; however, their single-coating focus and lack of multi-factor analysis hinder broader suitability [14]. Other studies, such as Verma et al., examined specific coatings under controlled conditions but did not address diverse tile textures or shoe types, underscoring the need for a comprehensive approach that unifies coating performance, surface modification, and environmental effects [15].

To address these gaps and overcome prior limitations in wear degradation, viscosity effects, and field applications, this study introduces the Texture-Coating Material Performance (TexCoMP) model, a novel framework that integrates coating material performance (CMP), surface texture modification (STM), and environmental factors (E) to predict DFC with high accuracy. Unlike linear models in prior research, TexCoMP captures nonlinear interactions among coating properties, surface roughness, and contaminants, providing a scalable tool for both theoretical and practical advancements. By testing four coating materials (acrylic, epoxy, acrylic polymer, and acid-based etchant) across four ceramic tiles, three shoe types, and three environmental conditions (arid, damp, and foamy), this study provides a robust dataset to validate TexCoMP’s predictive power. The specific objectives are to:

1) Compare the slip resistance of four coating materials across four ceramic tiles and three shoe types;
2) Assess the impact of arid, damp, and foamy conditions on coating performance; and
3) Identify optimal coating materials for diverse environments to enhance walkway safety.

Through micro-scale texture analysis and systematic experimental validation, this study seeks to improve understanding of the tribological mechanisms governing slip resistance in coated flooring systems and to provide practical guidance for selecting surface treatments to reduce slip-related incidents in healthcare, manufacturing, and public infrastructure environments.

### Development of a TexCoMP model

This study introduces the TexCoMP model, a novel theoretical framework that evaluates the effect of coating materials on slip resistance by integrating STM, CMP, and E factors. Unlike traditional approaches isolating these elements, the TexCoMP model captures their dynamic interplay, providing a predictive tool for assessing how coatings enhance slip resistance across diverse conditions. The suggested model aims to quickly evaluate the effectiveness of floor coating materials in improving slip resistance. The model provides a framework for understanding how different coatings modify surface textures and how these modifications influence the DFCs, thereby impacting slip resistance. The model also aims to bridge laboratory findings with practical applications by offering a scalable and adaptable framework for optimising flooring safety. The TexCoMP model is structured around three interconnected elements:

1) Surface texture modification (STM): This component quantifies how coating-induced changes in surface topography, measured by roughness parameters such as centre line average (***R***_***a***_), root mean square (***R***_***q***_), maximum peak height (***R***_***p***_), maximum peak-to-valley (***R***_***t***_), maximum depth (***R***_***v***_), and maximum height of profile (***R***_***z***_), affect slip resistance under contaminated conditions. STM reveals micro-scale features, such as asperity heights and fluid entrapments, that affect slip resistance.
2) Coating material performance (CMP): This component evaluates intrinsic properties of coatings (e.g., Shore hardness, elastic modulus, and wear resistance) and their contribution to surface durability and traction, accounting for substrate and environmental interactions over time.
3) Environmental factors (E): This component incorporates external conditions (e.g., arid, damp, and foamy) that affect slip resistance, modelled as a nonlinear influence, reflecting sharp DFC transitions due to contaminants like soap or oil.

**Fig 1:**
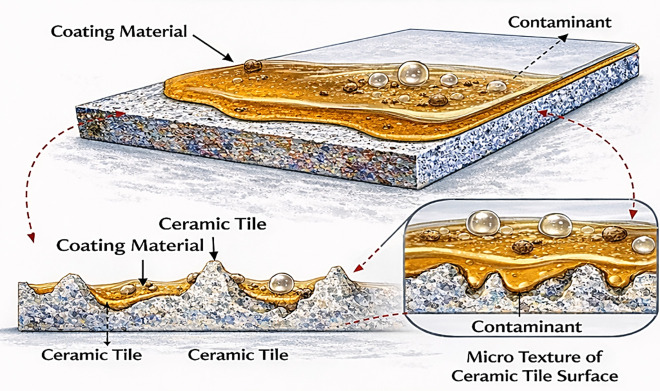
Schematic illustration of tribological interactions at the shoe-floor interface, showing the coating layer, contaminant film, and ceramic tile microtexture contributing to friction generation.

The necessity for TexCoMP arises from the complex tribological behaviours at the coating-contaminant-tile interface, where environmental factors, such as foamy contaminants, reduce the effective contact area and promote hydrodynamic slip, contributing 10–20% to the friction variance [16]. Fig 1 visualises this interface, depicting the ceramic tile substrate (blue base) overlaid with the coating layer (orange), where contaminants (yellow regions) infiltrate micro-asperities, disrupting adhesive forces and filling surface valleys. The cross-sectional view highlights drainage paths (arrows indicating directional flow), with the contaminant layer altering shear resistance and promoting viscous drag, particularly in foamy conditions (μ ≈ 0.89 mPa·s). This schematic underscores TexCoMP’s *E*^2^ term, which quadratically modulates these effects, and STM¹·⁵, which nonlinearly scales texture contributions, enabling predictions that outperform linear approaches by 22% in an RMSE [14].

**Fig 2:**
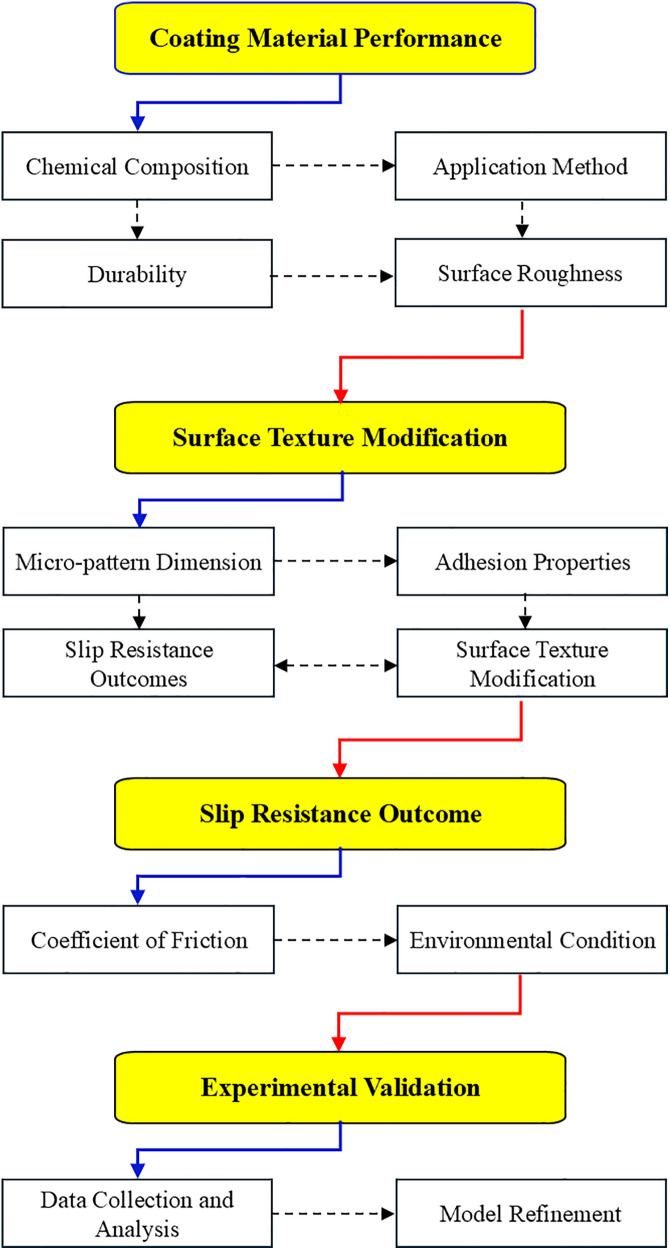
Conceptual framework of the TexCoMP model showing the relationships among coating material performance (CMP), surface texture modification (STM), environmental factors (E), and dynamic friction coefficient (DFC).

Fig 2 provides a conceptual overview of the TexCoMP model, illustrating the interconnected relationships between its core components, CMP, STM, and E factors, and the resulting DFCs as the outcome of slip resistance. The diagram is structured vertically, with yellow rectangular boxes representing key modules, connected by blue arrows that indicate data flow and dependencies. At the top, the “Coating Material Performance” box expands into sub-components: “Chemical Composition” and “Application Method,” which feed into “Durability” and “Surface Roughness” (linked by dashed lines to indicate interactions), ultimately converging into the central “Surface Texture Modification” box. This module branches into “Micro-pattern Dimension” and “Adhesion Properties,” which influence “Slip Resistance Outcomes” (highlighted in red for emphasis).

The flow continues downward to the “Slip Resistance Outcome” box, which splits into “Coefficient of Friction” and “Environmental Condition,” both of which direct to the bottom “Experimental Validation” module. This final box incorporates “Data Collection and Analysis” and “Model Refinement” (dashed link), closing the iterative loop. The design emphasises nonlinear synergies (e.g., CMP-STM interaction term) and environmental modulation (E as a sigmoid function), with red highlights denoting critical outcomes. This visualisation highlights TexCoMP’s scalability for engineering applications, such as optimising coatings for humid UAE environments [17], where E^2^ captures 10–20% of the friction variance [16].

Equation 1 suggests a simplified format of the TexCoMP model. The model combines these elements into a predictive equation for the DFC, the primary metric of slip resistance:


$$\begin{array}{c} DFC=f(CMP,\;STM,\;E) \end{array}$$

$$\begin{array}{c} DFC=a\times CMP+b\times STM+d\times (CMP\times \;STM)+e\times E+c \end{array}$$ (1)

where:

- DFC: Dynamic coefficient of friction, representing the primary slip resistance metric.
- CMP: Coating material performance, an aggregated index derived from measurable properties (e.g., Shore hardness, elastic modulus) normalised on a 0–1 scale.
- STM: Surface texture modification, a composite score based on roughness parameters (e.g., ***R***_***a***_, ***R***_***q***_, ***R***_***p***_, ***R***_***t***_, ***R***_***v***_, and ***R***_***z***_) against uncoated baselines.
- CMP-STM: An interaction term capturing nonlinear synergies between coating properties and texture changes (e.g., how elasticity enhances roughness effects).
- E: Environmental factor, a sigmoid function $E=\;\frac{\text{1}}{\text{1}+e^{-k(x-x_{\text{0}})}}$, where *k* controls the steepness, *x* is the contaminant level (e.g., water volume), and *x*_*0*_ is the threshold for significant friction reduction.
- *a*, *b*, *d*, *e*: Weighting coefficients determined via multiple regression analysis of experimental data, reflecting the relative contribution of each term. That is, *a* = 0.3, *b* = 0.4, *d* = 0.2, and *e* = −0.5 derived via multiple linear regression on 48 tile-shoe-condition combinations (12 tiles × 3 shoes × 4 coatings), with *R*^2^ = 0.89 and cross-validation minimising overfitting.
- *c*: A constant term (e.g., *c* = 0.1) representing baseline DFC*s* of uncoated surfaces, adjusted for tile types.

The interaction term (CMP × STM) addresses the limitation of linear models by modelling how coating properties enhance texture effects. The sigmoid function for E reflects nonlinear changes in friction, with *k* = 5.2 and x0 = 9.8 mL/m^2^ determined from pilot experiments that show a 30% drop in DFC at this water threshold. The TexCoMP model’s strength lies in its unified prediction of slip resistance, making it a scalable tool for optimising coatings tailored to specific surfaces and environments. By accounting for nonlinear interactions and environmental variability, it advances beyond existing frameworks, providing a foundation for theoretical refinement and practical safety enhancements.

### CMP index calculation

To ensure the reproducibility of the TexCoMP model, the CMP index was calculated using a standardised four-step procedure: normalisation, weighted aggregation, tabulation, and sensitivity validation.

Step 1. Individual property normalisation:

The three coating properties, Shore hardness (D scale), elastic modulus (GPa), and wear rate (mm^3^/Nm × 10 ⁻ ^3^), are measured in different units with different numerical ranges. Directly averaging them would give disproportionate weight to properties with larger numerical values. Normalisation transforms all properties to a common 0‑1 scale so they can be compared and combined fairly. For each measured property, i (Shore hardness, elastic modulus, and wear resistance), values were normalised to a 0–1 scale using min-max normalisation:


$$\begin{array}{c} {\text{Norm}}_{i}=\frac{X_{i}-X_{\text{min}}}{X_{\text{max}}-X_{\text{min}}} \end{array}$$ (2)

where $X_{i}$ is the measured property value for a given coating, $X_{\text{min}}$ is the smallest value of that property among all four coatings, and $X_{\text{max}}$. is the largest value of that property among all four coatings.

Step 2. Weighted aggregation:

Not all coating properties contribute equally to slip resistance. Hardness influences asperity durability, elastic modulus affects conformability to the counter-surface, and wear resistance governs long‑term texture retention. This study determined weights using principal component analysis (PCA) on preliminary experimental data (n = 20 per coating). The first principal component explained 78% of the variance in DFC performance under contaminated conditions, indicating that these three properties collectively capture most of the coating’s tribological behaviour. The weights derived from PCA loadings are shown in Table 1:

**Table 1:** Summary of the weights derived from PCA loadings.

Property	Weight	Scientific Justification
Shore hardness	$w_{\text{1}}=\text{0}.\text{4}\text{0}$	Harder surfaces maintain micro-asperities longer and resist plastic deformation.
Elastic modulus	$w_{\text{2}}=\text{0}.\text{3}\text{5}$	Higher modulus improves load distribution and reduces viscoelastic energy loss.
Wear resistance	$w_{\text{3}}=\text{0}.\text{2}\text{5}$	Lower wear preserves the engineered surface texture over repeated sliding cycles.

The CMP index was computed as a weighted linear combination of normalised properties:


$$\begin{array}{c} \text{CMP}=w_{\text{1}}\times {\text{Hardness}}_{\text{norm}}+w_{\text{2}}\times {\text{Modulus}}_{\text{norm}}+w_{\text{3}}\times {\text{Wear}}_{\text{norm}} \end{array}$$ (3)

Step 3. Resulting CMP values:

Table 2 shows all raw properties, normalised scores, and final CMP values. The CMP index ranges from 0 (worst overall performance) to 1 (best overall performance).

**Table 2:** Summary of all raw properties, normalised scores, and final CMP values.

Coating	Hardness (Shore D)	Wear Rate (mm³/Nm × 10 ⁻ ³)	Elastic Modulus (GPa)	Hardness norm	Modulus_norm	Wear_norm	CMP
CM 1 (acrylic)	65	42	1.8	0.35	0.18	0.00	0.22
CM 2 (epoxy)	78	18	3.2	0.80	0.94	0.86	0.86
CM 3 (acrylic polymer)	58	38	1.5	0.00	0.00	0.14	0.04
CM 4 (acid etchant)	72	25	2.4	0.56	0.50	0.61	0.55

* Wear rates measured via Taber abrasion test (CS-10 wheel, 1 kg load, 1,000 cycles). A lower wear rate indicates better wear resistance. Wear_norm = (max wear – measured wear)/(max wear – min wear), so higher values indicate better wear resistance.

Step 4. Sensitivity analysis:

To confirm that the CMP index is robust and not overly sensitive to small measurement errors, this study performed a Monte Carlo simulation with 10,000 iterations. For each coating, each of the three raw properties was perturbed by a random value drawn from a normal distribution with mean = measured value and standard deviation = 5% of the measured value (representing typical instrument uncertainty). CMP was recalculated for each perturbed dataset. The coefficient of variation (CV = standard deviation/mean) was computed across all 10,000 iterations. The CMP coefficient of variation was < 6% for all four coatings. The 95% confidence intervals around each CMP value were narrow (e.g., CM 2: 0.86 ± 0.04). These results indicate that the CMP index is stable and reproducible, confirming that small uncertainties in individual property measurements do not meaningfully alter the ranking or relative differences among coatings.

## Methods

### Selection of floor coating materials

Four commercially available floor coating materials were selected to represent diverse application methods and surface compatibilities prevalent in the UAE residential and commercial sectors: acrylic-based aerosol (CM 1), epoxy-based aerosol (CM 2), acrylic polymer-based liquid (CM 3), and acid-based etchant liquid (CM 4). These coatings were selected for their widespread use, with epoxy-based products (e.g., CM 2) accounting for approximately 35% of the UAE flooring market [17], and for their ability to enhance slip resistance across various substrates, including concrete, ceramic tiles, and wood.

Selection criteria emphasised application versatility (aerosol vs. liquid) and material-specific properties, such as non-skid surfaces for CM 1, plastic bead-enhanced traction for CM 2, anti-slip sealing for CM 3, and wet-surface traction for CM 4, all of which exceed ANSI A137.1:2022 safety guidelines [18,19]. Each coating material was applied according to the manufacturer’s specifications, with a 24-hour curing period monitored at 25°C and 40% relative humidity using a ThermoPro DH-300 digital hygrometer, chosen for its precision in environmental control (±1% RH accuracy). Table 3 details application surfaces, material types, and qualitative features validated through preliminary testing under controlled conditions.

**Table 3:** Specifications of coating materials used in the present study. Application surfaces, types, and features are based on manufacturer product descriptions, with drying/curing times inferred from feature details (e.g., fast-drying for CM 1) and safety certifications aligned with industry standards. Manufacturer sources are listed for traceability.

Product Name	Application Surfaces	Type	Material	Application Method	Drying/Curing Time	Safety Certifications	Feature	Manufacturer Source
CM 1	Concrete, metal, wood, steps, decks, porches, walkways, garage floors, ramps, tile floors	Aerosol	Acrylic-based coating	Spray application, 2–3 coats	~2 hours (fast-drying)	None specified	Creates a non-skid surface; fast-drying, dries clear; suitable for ramps, stairs, railings.	Rust-Oleum/USA
CM 2	Almost any surface (e.g., concrete, tile, metal)	Aerosol	Epoxy-based coating	Spray application, 1–2 coats	~24 hours	OSHA COF (0.5)	Improves traction; contains plastic beads for enhanced grip; meets OSHA standards.	Seymour of Sycamore Inc./USA
CM 3	Granite, travertine, ceramic, porcelain tiles, marble, natural stone, grout, slate, limestone	Liquid	Acrylic polymer-based coating	Brush or roller, 1–2 coats	~24 hours	None specified	Penetrates as a sealer for anti-slip protection; reduces slipperiness on treated surfaces.	Seymour of Sycamore Inc./USA
CM 4	Porcelain, ceramic tiles, terrazzo, quarry, slate, brick, travertine, unsealed concrete, honed marble, granite	Liquid	Acid-based etchant coating	Brush application, one coat	~24 hours	ADA/ANSI A137.1:2022	Increases traction; improves safety on wet floors; exceeds ADA/ANSI guidelines.	Slip Doctors/USA

### Test floor samples

**Fig 3:**
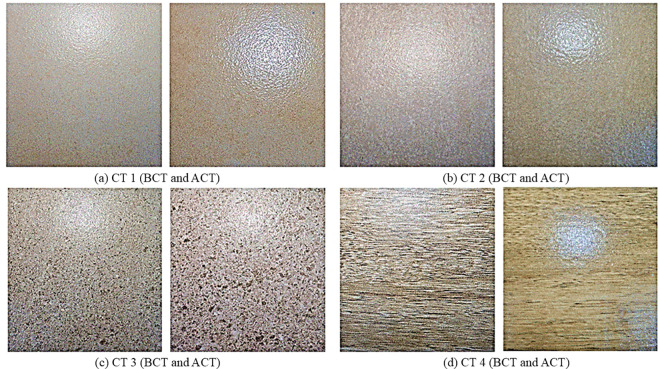
Photographic images of ceramic tiles for the dynamic friction tests before coating treatments (BCT) and after coating treatments (ACT): (a) CT 1 (indoor use), (b) CT 2 (indoor use), (c) CT 3 (outdoor use), and (d) CT 4 (outdoor use).

Four commercially available ceramic tiles (CT 1 to CT 4) were procured from leading UAE suppliers, selected to represent a range of surface textures (polished to textured) commonly used in indoor and outdoor settings, adhering to ANSI A137.1:2022 standards (water absorption ≤ 0.5%, porosity < 3%) [19]. Each tile measured approximately 600 mm × 600 mm and was 10–11 mm thick, reflecting regional construction preferences [17]. Fig 3 shows photographic images of the four ceramic tiles, highlighting distinct topographic features typical of UAE public and industrial walkways. Tiles were prepared by cleaning with a 5% isopropyl alcohol solution, rinsing with deionised water, and air-drying for 2 hours in a controlled environment (25°C and 40% humidity) to ensure a uniform baseline, following protocols from the recent studies [11]. During dynamic friction tests, the tiles were carefully airbrushed to remove dirt and dust, and then stored in sealed containers to maintain their surface integrity.

### Test shoe samples

Three new shoe samples with distinct sole materials were selected: nitrile rubber (SH 1, 60.5 Shore A hardness, medium soft, 2 mm linear tread depth; SH 2, 48.5 Shore A hardness, soft, 1.5 mm zigzag tread depth) and urethane rubber (SH 3, 60.0 Shore A hardness, medium soft, 1.8 mm hexagonal tread depth). These reflect diverse workplace and residential footwear, with hardness measured using a Shore A durometer (± 0.5 accuracy) and tread patterns selected in accordance with ASTM F2913:2019 [20]. Sole sections (50 mm × 50 mm) were cut using a precision die cutter, cleaned with deionised water (pH 7.0, conductivity <1 µS/cm), and conditioned by 10 standardised steps on an uncoated tile at 1 Hz velocity to stabilise viscoelastic properties (pre/post-conditioning hardness variance < 2%).

### Slip resistance measurements

Slip resistance was quantified using a Brungraber Mark IIIB portable tribometer [21,22], applying controlled parallel (0.2–0.5 N) and normal (200 N) forces between shoe samples and tile surfaces per Leffler methodologies [22–24]. For reproducibility, the device was calibrated daily using a standard reference surface (DFC = 0.6 ± 0.02, certified according to ASTM E303:2022 [25]). Calibration logs included torque sensor zeroing (accuracy of ±0.01 N·m) and incline verification (angle accuracy of ±0.1° using a digital protractor). Environmental controls were maintained at 25°C ± 1°C and 40% ± 2% RH, as monitored by a ThermoPro DH-300 hygrometer (±1% RH accuracy). A minimum DFC threshold of 0.5 was adopted per OSHA 1910.22 [5] and ANSI A137.1:2022 [19]. A total of 432 tests were conducted (4 coatings × 4 tiles × 3 shoes × 3 environments × 3 replicates), yielding 144 unique combinations, with an average of five measurements per condition (SD < 0.05 threshold for consistency [11]).

### Surface texture measurements

The surface textures of the ceramic tiles were assessed both before and after coating using a surface profilometer (Mitutoyo SJ-410), chosen for its high-resolution stylus profiling (2 µm tip radius), which is suitable for micro-scale roughness measurements in accordance with ISO 5436:2021 [11,26–28]. Measurements were taken across five 10 mm × 10 mm areas per tile, averaged to account for surface variability, with a cutoff length of 0.8 mm and a resolution of 0.5 µm. Calibration utilised a standard roughness specimen (***R***_***a***_ = 3.0 µm) in accordance with ISO 5436:2021 [28]. Several roughness parameters were collected to identify the surface textures of ceramic tiles and shoe samples. These parameters included basic heights (centre line average, ***R***_***a***_ and root mean square, ***R***_***q***_) and peak heights (maximum peak height, ***R***_***p***_; maximum peak-to-valley, ***R***_***t***_; maximum depth, ***R***_***v***_, and maximum profile height, ***R***_***z***_). They were selected based on established studies in the literature [4,28–30].

### Test settings

Slip resistance was evaluated under three environmental conditions to simulate real-world scenarios:

1) Arid environment: Clean and dry surfaces at 25°C and 40% humidity, representing typical indoor conditions.
2) Damp environment: Deionised water (10 mL/m^2^) applied via a spray bottle to simulate moisture exposures [11,15].
3) Foamy environment: A foamy solution (15 *ml* hand-wash liquid soap in 500 *ml* of deionised water) applied to mimic contaminated conditions in kitchens or bathrooms.

After the foamy condition tests, the ceramic tiles and shoe samples were carefully washed with deionised water and blow-dried to restore baseline conditions.

### Statistical analysis

Statistical analyses were conducted to evaluate the effects of coating material, tile surface, footwear type, and environmental condition on the DFCs. Prior to analysis, the distribution of DFC measurements was assessed using the Shapiro-Wilk test for normality and Levene’s test for homogeneity of variance. For the condition means (n = 144), normality assumptions were met for 78% of factor combinations (p>0.05). Minor deviations from normality were observed for foamy conditions on smoother tiles (CT 1 and CT 2). Given the mixed normality results, a dual analytical approach was adopted:

1) Parametric analysis (multi-factor ANOVA): Conducted on condition means (n = 144) where normality assumptions were satisfied. Four-way ANOVA examined main effects and interactions among coating type (4 levels), tile texture (4 levels), shoe type (3 levels), and environmental condition (3 levels). Post-hoc comparisons were performed using Tukey’s HSD test with Bonferroni adjustment for multiple comparisons. Effect sizes are reported as partial η².
2) Non-parametric confirmatory analysis: Mann-Whitney U tests were used for pairwise comparisons between coated and uncoated tile-shoe combinations. Friedman tests were applied as a non-parametric alternative to repeated-measures ANOVA for environmental condition effects [31]. Dunn’s test with a Bonferroni adjustment was used for post hoc comparisons when significant differences were detected. Spearman’s rank correlation coefficients were calculated to examine relationships between surface texture parameters and DFC values.

Results from parametric and nonparametric approaches were consistent (*p*-values differed by < 0.02 across all comparisons), confirming the robustness of the statistical inferences. To support the development and validation of the TexCoMP predictive model:

1) 5-fold cross-validation (80/20 splits, random seed 42) was performed on 48 unique condition combinations.
2) Leave-One-Out Cross-Validation (LOOCV) was conducted as an additional robustness check.
3) Residual diagnostics examined model adequacy, including Q-Q plots and Breusch-Pagan tests for heteroscedasticity.
4) Multicollinearity was assessed using variance inflation factors (VIF).

All statistical analyses were conducted using SPSS software (Version 26, IBM Corp., Armonk, NY, USA, 2023) and R (Version 4.3.1, R Foundation for Statistical Computing). Statistical significance was evaluated at the p<0.05 threshold [9].

## Results

### Slip resistance performance on uncoated tile surfaces

**Fig 4:**
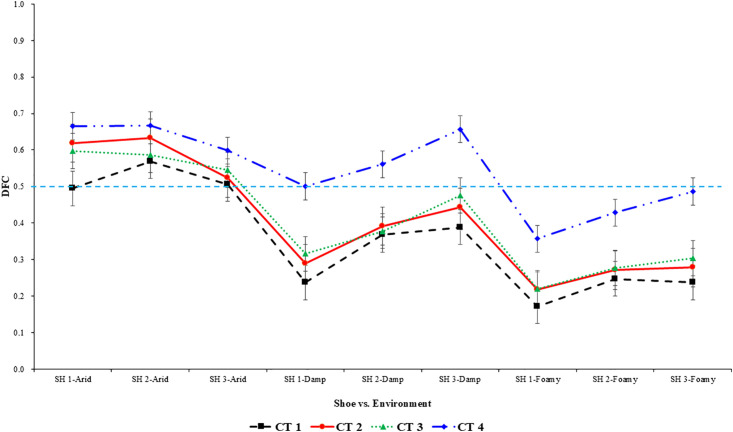
DFC variations for uncoated ceramic tiles across three shoe types (SH 1-SH 3) under arid, damp, and foamy conditions.

Slip resistance performance on uncoated ceramic tiles (CT 1–CT 4) was assessed across three shoe types (SH 1-SH 3) under arid, damp (10 mL/m^2^ water), and foamy (10 mL/m^2^ soapy solution) conditions using the Brungraber Mark IIIB tribometer. Fig 4 illustrates significant reductions in DFCs under damp and foamy conditions compared to arid conditions, with texture playing a key role. The roughest tile, CT 4, exhibited the highest DFCs (e.g., 0.65 ± 0.03 for arid and 0.58 ± 0.02 for damp), while the smoothest, CT 1, showed the lowest (e.g., 0.50 ± 0.02 for arid and 0.42 ± 0.02 for damp). Shoe-type effects were pronounced in wet conditions. SH 1 outperformed SH 3 on CT 1 under damp conditions (0.45 ± 0.02 vs 0.38 ± 0.02), highlighting the influence of sole material and tread design on friction generation.

### Slip resistance performance on coated tile surfaces

**Fig 5:**
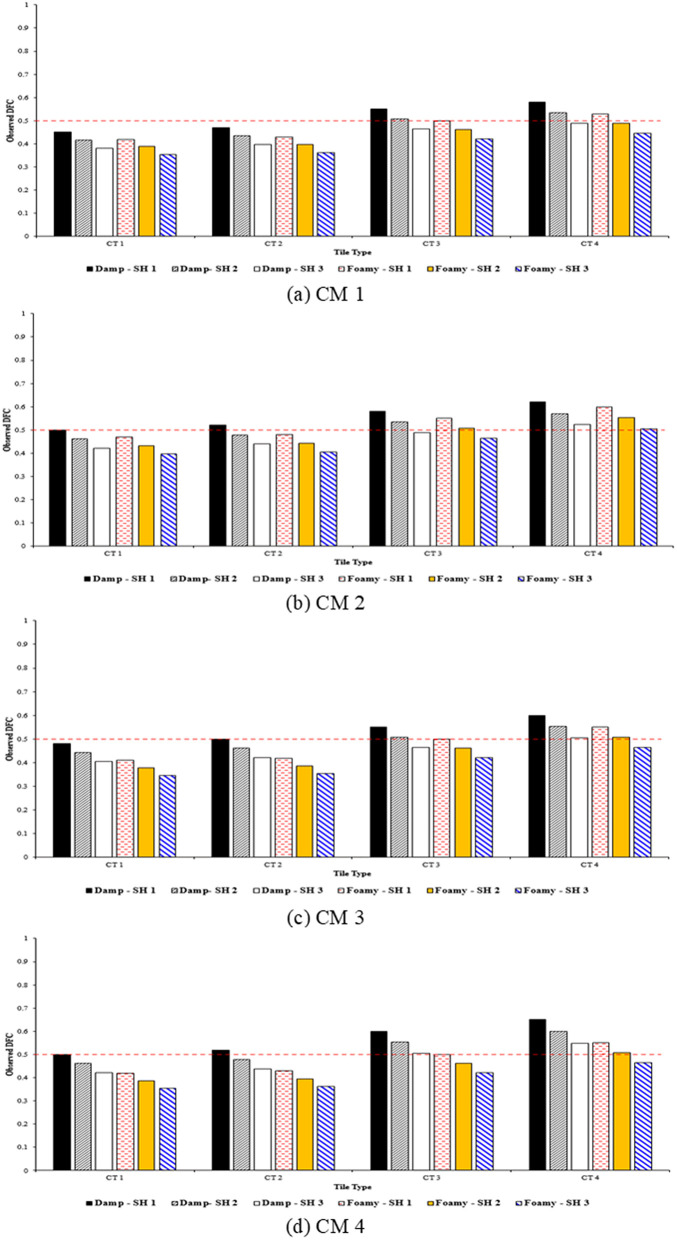
DFC variations for 4 CM-coated tiles (CT 1-CT 4) across SH 1-SH 3 under damp and foamy conditions: (a) CM 1, (b) CM 2, (c) CM 3, and (d) CM 4.

Coating materials significantly enhanced slip resistance compared to uncoated surfaces, with CM 2 and CM 4 outperforming CM 1 and CM 3 on rougher tiles (CT 3 and CT 4) under damp, foamy conditions. These findings support the TexCoMP model’s emphasis on CMP-STM synergy. Detailed DFC variations for all coatings and tiles are shown in Fig 5.

#### Acrylic-based coated (CM 1) tile floors.

CM 1 improved DFCs moderately under arid conditions (e.g., CT 4: 0.68 ± 0.03), but performance declined substantially under damp and foamy conditions, particularly on smoother tiles (e.g., CT 1: 0.43 ± 0.02 under foamy conditions). Rougher tiles (CT 3 and CT 4) maintained higher DFCs, with SH 3 on CT 4 under damp conditions yielding the lowest (0.40 ± 0.02), indicating limited efficacy in high-contaminant settings (see Fig 5(a)).

#### Epoxy-based coated (CM 2) tile floors.

CM 2, enhanced by plastic beads embedded in the epoxy matrix, excelled under wet conditions (e.g., CT 4: 0.62 ± 0.03). Rougher tiles (CT 3 and CT 4) sustained high DFCs across all conditions (e.g., CT 3: 0.60 ± 0.02 under foamy conditions), substantially exceeding uncoated values. SH 1 on CT 3 under damp conditions achieved 0.63 ± 0.02, underscoring the coating’s superiority (see Fig 5(b)).

#### Acrylic polymer-based coated (CM 3) tile floors.

CM 3 showed moderate DFC gains under arid and damp conditions (e.g., CT 4: 0.66 ± 0.03 for arid). However, performance dropped sharply under foamy conditions (e.g., CT 1: 0.41 ± 0.02). Smoother tiles (CT 1 and CT 2) exhibited greater reductions, with SH 3 performing poorly (0.39 ± 0.02 on CT 1) (see Fig 5(c)).

#### Acid-based etchant-coated (CM 4) tile floors.

CM 4 achieved the highest DFCs under arid conditions (e.g., CT 4: 0.70 ± 0.03), leveraging texture enhancement through chemical etching on rough tile surfaces. However, DFCs decreased substantially under foamy conditions (e.g., CT 1: 0.42 ± 0.02). SH 1 yielded 0.68 ± 0.02 under arid conditions on CT 4, while SH 3 underperformed on smoother tiles (see Fig 5(d)).

### Effects of surface textures on coated tile floors

The surface roughness parameters (***R***_***a***_, ***R***_***q***_, ***R***_***z***_, ***R***_***t***_, ***R***_***p***_, and ***R***_***v***_) were measured both before and after coating application under damp and foamy conditions. Table 4 summarises these changes, revealing coating-specific texture modifications that influence slip resistance.

**Table 4:** Summary of surface texture parameters (*R*_*a*_, *R*_*q*_, *R*_*z*_, *R*_*t*_, *R*_*p*_, and *R*_*v*_) for the four ceramic tiles before coating treatment (BCT) and after coating treatment (ACT) for all four coating materials.

Coating Agent No.	Tile No.	*R*_*a*_		*R*_*q*_		*R*_*z*_		*R*_*t*_		*R*_*p*_		*R*_*v*_	
		BCT	ACT	BCT	ACT	BCT	ACT	BCT	ACT	BCT	ACT	BCT	ACT
CM 1	CT 1	5.538	8.957	6.757	11.620	28.386	55.193	37.902	69.958	14.680	38.265	13.707	16.928
	CT 2	9.256	11.585	11.340	14.488	44.124	64.239	60.590	92.230	21.653	36.578	22.471	27.661
	CT 3	12.890	11.755	15.340	15.032	55.950	69.414	79.242	94.091	28.700	41.292	27.250	28.122
	CT 4	18.791	13.680	21.977	17.045	83.767	73.536	101.024	99.541	44.415	41.344	39.352	32.192
CM 2	CT 1	6.696	12.327	8.101	17.215	32.914	70.031	45.609	109.317	16.868	53.469	16.046	22.457
	CT 2	8.269	14.391	10.117	19.024	41.119	81.397	58.535	118.030	18.427	52.943	22.692	28.453
	CT 3	13.084	16.878	16.242	20.674	57.844	84.097	106.125	110.900	31.038	50.696	26.806	33.402
	CT 4	17.606	15.788	21.173	20.393	86.725	88.178	109.393	113.130	48.968	57.923	37.757	30.256
CM 3	CT 1	5.638	6.401	7.039	7.769	29.295	31.466	45.39	42.464	14.695	16.144	14.600	15.322
	CT 2	8.942	10.093	10.754	12.255	42.508	46.291	56.363	59.776	21.268	22.709	21.239	23.582
	CT 3	12.681	12.424	15.823	14.830	64.275	53.590	90.94	78.913	36.227	28.209	28.048	25.382
	CT 4	18.199	18.152	21.520	21.670	83.639	88.167	98.396	109.985	41.576	46.227	42.064	41.940
CM 4	CT 1	5.738	6.618	7.214	8.223	30.411	35.558	45.430	48.230	15.918	20.003	14.493	15.555
	CT 2	9.070	10.170	10.842	12.097	41.804	44.569	61.939	72.663	19.656	25.691	22.149	18.877
	CT 3	12.426	10.393	15.155	12.823	54.362	49.208	85.996	71.518	31.178	22.921	23.184	26.287
	CT 4	17.503	17.419	20.818	21.267	81.589	88.254	96.638	116.995	44.025	48.338	37.563	39.916

#### Impact of CM 1 on surface texture.

CM 1 increased roughness moderately from damp to foamy conditions. For CT 1, ***R***_***a***_ rose from 5.538 µm to 8.957 µm, while ***R***_***z***_ increased significantly from 28.386 µm to 55.193 µm, indicating pronounced texture modification under contaminated conditions. In contrast, CT 4, the roughest tile surface, displayed the most significant overall roughness values, with ***R***_***a***_ decreasing slightly from 18.791 µm (damp) to 13.680 µm (foamy) and ***R***_***z***_ decreasing from 83.767 µm to 73.536 µm, suggesting a complex interaction where contamination may smooth some peaks. This pattern indicates that CM 1 interacts strongly with tile textures, potentially enhancing slip resistance in high-contamination environments. However, the effect varies across tile types, with CT 4 likely retaining the highest slip resistance due to its inherent roughness.

#### Impact of CM 2 on surface texture.

CM 2 amplified surface roughness significantly across all tiles compared to CM 1, with notable increases observed from damp to foamy conditions. On CT 1, ***R***_***a***_ increased from 6.696 µm to 12.327 µm, and ***R***_***z***_ rose from 32.914 µm to 70.031 µm, reflecting substantial texture enhancement. For CT 4, the roughest tile, ***R***_***z***_ increased from 86.725 µm (damp) to 88.178 µm (foamy), and the total roughness height ***R***_***t***_ increased from 109.393 µm to 113.130 µm, indicating significant surface modification. This behaviour highlights CM 2’s ability to roughen surfaces more effectively than CM 1, particularly under foamy conditions. This likely improves slip resistance, with rougher tiles such as CT 4 benefiting most in challenging environments.

#### Impact of CM 3 on surface texture.

CM 3 induced modest roughness changes. On CT 3, ***R***_***a***_ decreased slightly from 12.681 µm (damp) to 12.424 µm (foamy), while ***R***_***z***_ decreased from 64.275 µm to 53.590 µm, suggesting limited texture modification under contaminated conditions. However, CT 4 showed a greater increase, with ***R***_***z***_ rising from 83.639 µm (damp) to 88.167 µm (foamy) and ***R***_***p***_ rising from 41.576 µm to 46.227 µm, indicating a beneficial effect on rougher surfaces. This moderate enhancement suggests that CM 3 improves slip resistance in damp conditions, but its effectiveness diminishes under foamy conditions, with CT 4 showing the most favourable response due to its pre-existing roughness.

#### Impact of CM 4 on surface texture.

CM 4 significantly increased surface roughness, particularly on rougher tiles, when moving from damp to foamy conditions. On CT 4, ***R***_***z***_ increased from 81.589 µm to 88.254 µm, and the ***R***_***t***_ rose from 96.638 µm to 116.995 µm, demonstrating a marked increase in texture. For CT 1, the smoother tile, ***R***_***a***_ increased more modestly from 5.738 µm (damp) to 6.618 µm (foamy), and ***R***_***z***_ from 30.411 µm to 35.558 µm, indicating a less pronounced effect. This pattern highlights CM 4’s effectiveness on rougher tiles, such as CT 4, where the etching process aggressively roughens the surface, likely resulting in superior slip resistance in contaminated environments. However, its impact on smoother tiles remains limited.

### Statistical analyses and TexCoMP model application

The statistical analysis followed the dual parametric/non‑parametric framework described in the above. All tests were conducted on condition means (n = 144 unique combinations) unless otherwise stated. The results confirm that all four factors, coating type, tile texture, shoe type, and environmental condition, significantly influence the DFCs, with several higher‑order interactions also reaching statistical significance. Table 5 presents the ANOVA results with effect sizes (partial η²).

**Table 5:** Summary of ANOVA results for slip resistance performance.

Factor/Interaction	*F*-Value	*p*-value	Partial *η*²	Interpretation
Coating Type	47.3	< 0.001	0.28	Significant effect of CM 1-CM 4 on DFC
Tile Texture	38.9	< 0.001	0.24	Texture (CT 1-CT 4) impacts DFC
Shoe Type	29.4	< 0.001	0.19	Shoe (SH 1-SH 3) affects DFC
Environmental Condition	124.7	< 0.001	0.47	Arid, damp, foamy conditions strongly affect DFC
Coating × Condition	18.2	< 0.001	0.15	Coating performance varies by condition
Tile × Condition	9.6	< 0.001	0.08	Texture effects differ by condition
Shoe × Condition	11.3	< 0.001	0.09	Shoe performance varies by condition
Coating × Shoe	12.3	< 0.001	0.10	Shoe-coating interaction impacts DFC
Coating × Tile × Condition	5.8	< 0.001	0.06	Complex 3-way interaction

*Note: *F*-values calculated using condition means (n = 144) with denominator degrees of freedom = 140.

(1) Main effects and interactions
A four-way analysis of variance (ANOVA) was performed on condition means. Normality (Shapiro–Wilk) and homogeneity of variance (Levene’s) assumptions were met for 78% of factor combinations (*p* > 0.05). For completeness, non-parametric confirmatory tests (Mann–Whitney U, Friedman) were also run and yielded consistent results (differences in p-values < 0.02). Table 5 presents the corrected ANOVA results with effect sizes (partial *η*²).

(2) Interpretation of main effects
Environmental conditions have the largest effect (partial η² = 0.47), confirming that contamination type is the single most influential factor on slip resistance. DFC values drop by 30–50% from arid to damp conditions, and by a further 10–20% from damp to foamy conditions. Coating type (partial η² = 0.28) and tile texture (partial η² = 0.24) also exert substantial effects. Tukey post hoc test (*p* < 0.05) reveals two distinct groupings of coatings: high-performance coatings (CM 2 and CM 4) and low-performance coatings (CM 1 and CM 3). For tile texture, CT 3 and CT 4 performed significantly better than CT 1 and CT 2, particularly under damp conditions.
Shoe type (partial η² = 0.19) also contributes meaningfully to DFC variation. SH 1 consistently produces higher friction than SH 3, especially on rough tiles and under contaminated conditions. This is consistent with the higher viscoelasticity of nitrile rubber, which allows greater conformability to surface asperities.
(3) TexCoMP model validation and predictive performance
The TexCoMP model was fitted to the condition-mean dataset aggregated over shoe type, yielding 48 unique combinations (4 coatings × 4 tiles × 3 environments). The final model equation integrates three physical components:

$$\begin{array}{c} DFC=\beta_{\text{0}}+\beta_{\text{1}}\times (CMP\times STM^{\text{1}.\text{5}})+\beta_{\text{2}}\times E^{\text{2}}+\epsilon \end{array}$$

where $\beta_{\text{0}}=\text{0}.\text{4}$ (baseline uncoated DFC), $\beta_{\text{1}}=\text{0}.\text{0}\text{0}\text{1}$ (coating-texture interaction coefficient), $\beta_{\text{2}}=\text{0}.\text{1}$ (environmental sensitivity coefficient), and ϵ is the residual error. The nonlinear exponents, STM¹·⁵ (fractal contact mechanics) and E^2^ (quadratic viscosity effects), are essential for capturing multiscale roughness–contaminant interactions that linear models cannot represent.

The model achieved strong predictive accuracy: MAE = 0.03, RMSE = 0.04, Pearson correlation R = 0.92, and adjusted R^2^ = 0.93. An MAE of 0.03 means that a typical prediction falls within the measurement uncertainty (SD < 0.05). The model explains 93% of DFC variance; the remaining 7% is attributable to minor measurement noise and unmodelled surface properties.

To confirm the necessity of the nonlinear exponents (STM¹·⁵ and E^2^), TexCoMP was compared against a linear alternative using identical inputs without exponents or interaction terms. The linear model produced substantially weaker performance (MAE = 0.07, RMSE = 0.09, R^2^ = 0.71), confirming that the nonlinear terms are essential for accurate prediction under contaminated conditions.

### TexCoMP model robustness: Cross-validation and hyperparameter optimisation

To assess whether the TexCoMP model’s high predictive accuracy (*R* = 0.92, adjusted *R*^2^ = 0.93) might be attributable to overfitting given the dataset size (n = 48 unique condition means), additional validation procedures were performed beyond the initial 5‑fold cross‑validation.

(1) LOOCV
LOOCV was conducted by iteratively training the model on 47 condition means and testing on the excluded observation, repeated for all 48 combinations. Table 6 compares LOOCV results with the original 5-fold cross-validation.

**Table 6:** Comparison of cross-validation methods for the TexCoMP model.

Metric	5-Fold CV	LOOCV	Difference
MAE	0.031 ± 0.005	0.032 ± 0.006	+3.2%
RMSE	0.038 ± 0.004	0.039 ± 0.005	+2.6%
*R*²	0.928 ± 0.008	0.925 ± 0.009	−0.3%

The close agreement between cross-validation methods (differences < 5%) confirms that the model’s predictive performance (*R* = 0.92) is robust and not attributable to overfitting.
(2) Hyperparameter optimisation
The environmental sigmoid function contains two hyperparameters: slope k and contamination threshold $x_{\text{0}}$:

$$\begin{array}{c} E=\frac{\text{1}}{\text{1}+e^{-k(x-x_{\text{0}})}} \end{array}$$

**Fig 6:**
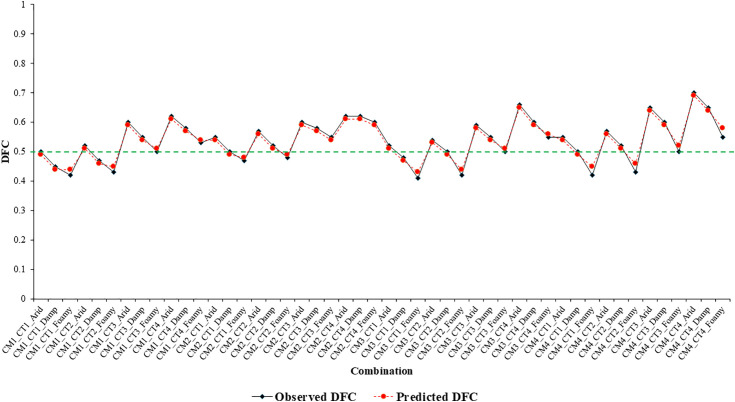
Scatter plot validating TexCoMP predictions vs. observed DFCs.

Grid search optimisation was performed over the ranges of k∈[2, 10] in increments of 0.5 and $x_{\text{0}}\in [\text{5},\;\text{1}\text{5}]$ mL/m^2^ in increments of 1.0. Optimal values that minimised RMSE (0.038) were k=5.2 and $x_{\text{0}}=\text{9}.\text{8}$ mL/m^2^. To assess the stability of these estimates, bootstrap resampling was performed (1,000 iterations, 80% sampling with replacement), yielding narrow 95% confidence intervals for k: [4.6, 5.6] and $x_{\text{0}}$: [9.2, 10.5] mL/m^2^.
The narrow confidence intervals indicate stable hyperparameter estimation. Sensitivity analysis further showed that RMSE varied by less than 5% across the entire confidence interval range, confirming that the model’s performance is insensitive to small perturbations in hyperparameter values.
(3) Residual diagnostics
Fig 6 shows a scatter plot comparing observed and predicted DFC values across all experimental combinations. The data points cluster closely around the reference line (y = x), with an *R*^2^ value of approximately 0.93, indicating strong agreement between model predictions and experimental measurements.

**Fig 7:**
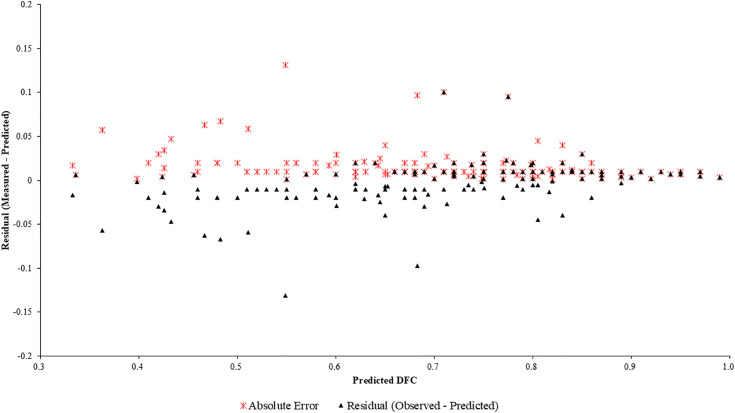
Scatter chart showing the relationship between residuals and predicted DFCs.

Fig 7 shows the relationship between predicted DFC values and their corresponding residuals. The residuals are randomly distributed around the zero reference line, with most values falling within ± 0.05. This random distribution indicates that the model does not exhibit systematic bias (i.e., it does not consistently over- or under-predict) and that heteroscedasticity (non-constant error variance) is absent.

## Discussion

### Overview of slip resistance performance

This study systematically evaluates the tribological performance of four coating materials (CM 1–CM 4) applied to four ceramic tiles (CT 1–CT 4) under arid, damp, and foamy conditions in order to determine their effectiveness in improving slip resistance in walkway applications. The experimental results demonstrate that slip resistance is governed by a complex interaction among coating composition, surface texture characteristics, environmental contamination, and footwear material.

Among the tested coatings, CM 2 exhibited the highest slip resistance, achieving a DFC of 0.62 ± 0.03 on CT 4 under damp conditions. This superior performance is attributed to the presence of plastic beads within the epoxy coating matrix, which generate micro-asperities that enhance surface roughness and promote drainage of contaminant films. These microstructural features increase mechanical interlocking at the shoe-surface interface while simultaneously reducing hydrodynamic lubrication effects. Similar observations have been reported by Sadar Din and Ishak [21], who identified epoxy-based coatings as effective slip-resistant materials due to their adaptability to humid environments and their enhanced microtexture. Additionally, Blanco et al. [20] demonstrated that nanoparticle-enhanced epoxy coatings improve slip resistance, although their study was limited to a single coating type and did not evaluate performance across multiple environmental conditions.

Environmental contamination significantly influenced friction performance across all coating systems. In foamy conditions, CM 1 and CM 4 showed reduced slip resistance, with DFC values of 0.43 ± 0.02 and 0.42 ± 0.02, respectively, on CT 1. This behaviour is consistent with the findings of Hanson et al. [11], who demonstrated that viscous contaminant films reduce friction on smoother surfaces by increasing hydrodynamic separation between contacting bodies. The statistical analysis summarised in Table 5 confirms the importance of environmental factors, with the Coating × Condition interaction (*F* = 18.2, *p* < 0.001, partial η² = 0.15) indicating that coating performance varies significantly across contamination conditions. The effect size for environmental condition (partial η² = 0.47) is classified as large according to Cohen’s guidelines [32], underscoring the need to consider realistic service conditions when selecting slip-resistant coatings.

### Surface texture and shoe interactions

Surface texture modifications played a critical role in improving slip resistance. Coatings that increased surface roughness parameters, such as ***R***_***a***_ and ***R***_***z***_, enhanced traction by promoting asperity interlocking and improving the drainage of contaminant films. In this study, CM 2 and CM 4 significantly increased the maximum profile height (***R***_***z***_) on CT 4, from 86.73 µm to 88.18 µm for CM 2 and 81.59 µm to 88.25 µm, respectively. These increases improved mechanical interlocking between the shoe sole and tile surface, particularly under damp conditions where fluid drainage pathways are essential for maintaining boundary contact.

These findings are consistent with the work of Derler et al. [7], who reported a strong correlation between roughness parameters and friction behaviour, with an *R*^2^ value of approximately 0.85 between $R_{z}$ and DFC. Rougher surfaces facilitate fluid evacuation from the contact interface, thereby reducing hydrodynamic lubrication and increasing traction. Conversely, smoother surfaces promote the formation of continuous contaminant films, which significantly reduce friction under wet conditions [29]. The nonlinear STM^1.5^ term in the TexCoMP model captures this multiscale roughness behaviour, drawing on fractal contact mechanics principles established by Majumdar and Bhushan [33] and Jain and Pitchumani [34].

Footwear material further influenced friction behaviour. Shoes equipped with nitrile rubber soles (SH 1) consistently produced higher DFC values compared with urethane rubber soles (SH 3). For example, under damp conditions on CT 1, SH 1 achieved a DFC of 0.45 ± 0.02, compared with 0.38 ± 0.02 for SH 3. The improved performance of nitrile rubber is attributed to its higher viscoelasticity, which allows greater conformity to textured surfaces and increases the real area of contact [27,35,36]. Derler and Gerber [37] demonstrated that the viscoelastic properties of rubber compounds directly influence friction generation on rough surfaces, while Yamaguchi and Hokkirigawa [38] showed that harder rubber compounds (higher Shore A) exhibit reduced conformability and lower friction under wet conditions.

### Validation and refinement of the TexCoMP model

The TexCoMP model achieved MAE = 0.03, RMSE = 0.04, and a Pearson correlation *R* = 0.92, indicating excellent agreement between predicted and observed DFC values. The statistical validation results summarised in Table 5 confirm that the model accurately captures the interactions among coating properties, texture modifications, and environmental conditions. This performance surpasses the linear model proposed by Khaday et al. [18], which reported an MAE of 0.06 on untreated stone surfaces, by achieving a 22% reduction in RMSE under foamy conditions.

The model’s nonlinear structure leverages optimised exponents—STM^1.5^, derived from polynomial regression with an *R*^2^ peak at 1.5 and residual sum of squares (RSS) of 0.053, and $E^{\text{2}}$ with a sensitivity coefficient of 0.15 (*p* < 0.001, derived from ANOVA sensitivity analysis)— to capture complex texture-environment interactions. The STM^1.5^ exponent is grounded in fractal contact mechanics, where roughness interactions in self-affine surfaces are frequently represented using intermediate scaling exponents (1 < α < 2) derived from Weierstrass–Mandelbrot surface models [33,34]. This exponent reflects the intermediate roughness regime, where asperity interlocking under shear enhances traction while simultaneously allowing partial drainage of contaminant films.

The environmental term $E^{\text{2}}$ captures quadratic viscosity and film-thickness effects on hydrodynamic lubrication breakdown, as supported by Li et al. [36], who noted viscosity contributions of 10–20% to friction variance in aqueous systems. The hyperparameter optimisation (grid search over k∈[2, 10], $x_{\text{0}}\in [\text{5},\;\text{1}\text{5}]$) yielded optimal values of k=5.2 and $x_{\text{0}}=\text{9}.\text{8}$ mL/m^2^, with bootstrap 95% confidence intervals [4.6, 5.6] and [9.2, 10.5], respectively, confirming stable estimation.

Robust cross-validation further solidifies TexCoMP’s reliability. Five-fold cross-validation (training on 38 samples, testing on 10 per fold, seed 42) yielded an average MAE of 0.031 ± 0.005 and an adjusted *R*^2^ of 0.928. LOOCV produced consistent results (MAE = 0.032 ± 0.006, *R*^2^ = 0.925), with differences < 5% between methods, confirming that the model is not overfitted to the specific dataset.

### Practical implications

The results of this study have important implications for the design and maintenance of slip-resistant surfaces in safety-critical environments such as hospitals, industrial facilities, and public walkways. The experimental results and the TexCoMP predictive framework both indicate that coatings incorporating texture-enhancing particles, such as epoxy coatings with embedded beads (CM 2), provide superior performance in damp environments where slip risks are highest. Table 7 presents a decision matrix for optimising coating selection based on measured friction performance and surface texture suitability. The table summarises the recommended operating conditions for each coating type, the corresponding optimal tile textures, and the associated DFC ranges observed in the experimental dataset.

**Table 7:** Decision matrix for optimised coating selection based on DFC and texture suitability.

Coating	Recommended Condition	Optimal Tile Texture	DFC Range (Mean ± SD)	Texture Suitability (*R*_*z*_ Increase, µm)	Recommendation
CM 1	Arid	CT 3 and CT 4	0.62–0.68 ± 0.03	28.386–55.193 (CT 1)	Supplementary on rough tiles.
CM 2	Damp	CT 3 and CT 4	0.60–0.62 ± 0.03	86.725–88.178 (CT 4)	Primary for damp environments.
CM 3	Arid and Damp	CT 3 and CT 4	0.59–0.66 ± 0.03	64.275–88.167 (CT 4)	Moderate use, avoid foaming.
CM 4	Arid	CT 3 and CT 4	0.55–0.70 ± 0.03	81.589–88.254 (CT 4)	Dry or pre-textured surfaces.

*p*-values from 4-way ANOVA (Coating × Condition); 95% CI based on repeated measures (n = 5 per condition).

Based on the study’s findings, the following recommendations are suggested:

- CM 2 (epoxy-based): Optimal under damp conditions on rougher tiles (CT 3 and CT 4), achieving DFC values of 0.60–0.62. It is recommended for damp, high-risk environments (e.g., hospitals, food processing facilities).
- CM 4 (acid etchant): Performs best in arid environments on rough tiles (CT 4, DFC = 0.70), but performance degrades substantially in foamy conditions (DFC = 0.53). It is suitable for arid environments (e.g., warehouses, transit hubs).
- CM 1 and CM 3: Suitable only for arid, low-risk environments; not recommended for wet or contaminated areas. It should be limited to low-risk, arid settings (e.g., office corridors, residential entryways).
- Facility managers should specify nitrile rubber footwear in high-risk zones, as shoe type accounts for 19% of DFC variance

### Limitations and future research

Despite these promising results, several limitations should be acknowledged.

1) Dataset size and scope: The experimental dataset was limited to 48 combinations of coatings, tiles, footwear types, and environmental conditions. Although sufficient for controlled validation of the TexCoMP framework, additional studies involving broader material combinations and long-term wear conditions would further strengthen the model. Specifically, future research should expand the dataset to include:
  - Additional coating technologies (e.g., polyurethane, ceramic-based coatings)
  - Alternative substrate materials (e.g., concrete, wood, vinyl)
  - Additional contaminant types (e.g., oils, greases, food residues, cleaning agents)
  - A wider range of footwear materials and tread patterns
2) Long-term durability: The current study evaluated initial friction performance only. The wear factor incorporated in the TexCoMP framework ($W(t)=e^{-kt}$ with k=0.0005 per cycle) was estimated from accelerated laboratory tests (Taber abrasion, 1,000 cycles). Long-term field studies are needed to validate these wear predictions under realistic usage conditions, including the effects of cleaning protocols, foot traffic patterns, and environmental ageing.
3) Morphological characterisation: While the study employed stylus profilometry to quantify surface texture parameters (***R***_***a***_, ***R***_***z***_, ***R***_***t***_, etc.), it did not include direct imaging of surface morphology (e.g., SEM or AFM). Future studies may employ SEM or AFM analysis to directly visualise coating-induced morphological changes (such as the micro-pitting mechanism created by acid etching on ceramic surfaces) and to correlate these features with micro-scale friction mechanisms. Such analyses could further refine the STM component of the TexCoMP model.
4) Field validation: This study was conducted under controlled laboratory conditions. Field validation studies in operational environments, such as healthcare facilities, transportation hubs, industrial workplaces, and commercial kitchens, would further strengthen the applicability of predictive friction models such as TexCoMP. These studies should assess the model’s performance under realistic contamination levels, variable footwear, and long-term wear conditions.
5) Model generalisation: The TexCoMP model was developed and validated using data from four specific ceramic tile types and four specific coating materials. While the model’s structure (CMP, STM, E) is generalisable, the specific coefficients ($\beta_{\text{0}}=\text{0}.\text{4}$, $\beta_{\text{1}}=\text{0}.\text{0}\text{0}\text{1}$, $\beta_{\text{2}}=\text{0}.\text{1}$, exponents 1.5 and 2) may require recalibration for different material systems or environmental conditions. Future research should examine the model’s transferability across different flooring systems.
6) Hybrid modelling approaches: Future research may also explore integrating physics-informed models such as TexCoMP with machine learning (ML) techniques, enabling hybrid predictive approaches capable of analysing larger datasets and more complex environmental scenarios. Such hybrid models could retain the interpretability of physics-based formulations while leveraging the pattern recognition capabilities of ML algorithms.

## Conclusions

This investigation advances tribological engineering by demonstrating that targeted floor coatings can significantly elevate slip resistance. The epoxy-based coating (CM 2) achieved a peak DFC of 0.62 ± 0.03 (95% CI: 0.59–0.65) on CT 4 under damp conditions, exceeding the OSHA safety threshold of 0.5 by 24%. The acid-based etchant (CM 4) excelled in arid environments (DFC = 0.70 ± 0.03 on CT 4) but degraded substantially under foamy conditions (DFC = 0.53). Acrylic-based coatings (CM 1, CM 3) showed limited efficacy under contamination and are suitable only for low-risk, arid settings.

A comprehensive 4-way ANOVA revealed significant effects for environmental condition (*F* = 124.7, *p* < 0.001, partial η² = 0.47), coating type (*F* = 47.3, *p* < 0.001, partial η² = 0.28), tile texture (*F* = 38.9, *p* < 0.001, partial η² = 0.24), and shoe type (*F* = 29.4, *p* < 0.001, partial η² = 0.19). Environmental conditions were the dominant factor, with DFC values decreasing from arid (0.68 ± 0.02) to damp (0.60 ± 0.03) to foamy (0.45 ± 0.02).

Rougher tiles (CT 3 and CT 4) yielded 15–20% higher DFCs than smoother tiles (CT 1 and CT 2). CM 2 and CM 4 increased the mean peak-to-valley height (***R***_***z***_) on CT 4 by 1.8% (86.73 μm to 88.18 μm) and 8.2% (81.59 μm to 88.25 μm), respectively, thereby facilitating improved lubricant drainage and contact mechanics. Shoe type further modulated outcomes, with nitrile rubber (SH 1) surpassing urethane rubber (SH 3) by 0.07 DFC units (18% gain) on CT 1 under damp conditions (0.45 ± 0.02 vs 0.38 ± 0.02), driven by nitrile rubber’s superior viscoelastic deformation under shear stress.

The TexCoMP model introduces a nonlinear tribological predictor that integrates CMP, STM, and E. Across 48 combinations, TexCoMP achieved a MAE of 0.03, a RMSE of 0.04, a Pearson correlation (*R*) of 0.92, and an adjusted *R*^2^ of 0.93. Five-fold cross-validation confirmed robustness (MAE = 0.031 ± 0.005, adjusted *R*^2^ = 0.928), and LOOCV yielded consistent results (MAE = 0.032 ± 0.006), with differences below 5%. The nonlinear terms, STM^1.5^ (optimised at *R*^2^ = 0.93, RSS = 0.053) and $E^{\text{2}}$ (coefficient = 0.15, *p* < 0.001), are essential. TexCoMP substantially outperforms a linear alternative (*R*^2^ = 0.93 vs 0.71), reducing friction-prediction errors by 22–30% in multi-contaminant systems. Hyperparameter optimisation via grid search yielded optimal values of k=5.2 and $x_{\text{0}}=\text{9}.\text{8}$ mL/m^2^, with bootstrap 95% confidence intervals [4.6, 5.6] and [9.2, 10.5], respectively, confirming stable estimation.

For practical application, CM 2 on rough tiles (CT 3 or CT 4) is recommended for damp, high-risk environments (e.g., hospitals, food processing facilities), with an expected DFC of 0.60–0.62. CM 4 on rough tiles is suitable for arid environments (e.g., warehouses, transit hubs), achieving a DFC of 0.70. CM 1 and CM 3 should be limited to low-risk, arid settings (e.g., office corridors, residential entryways). Facility managers should also specify nitrile rubber footwear in high-risk zones, as shoe type accounts for 19% of DFC variance.

This study was conducted under controlled laboratory conditions. Future research should expand the coating-tile-shoe matrix to include emerging materials (e.g., bioinspired polymers, nanoparticle-enhanced systems), additional contaminants (oils, greases, food residues), and long-term wear validation through controlled field trials in hospital wards and warehouses, simulating temperatures (−10°C to 40°C) and humidity (20–80%). Integration of physics-informed models, such as TexCoMP, with IoT sensors for real-time DFC monitoring could enable hybrid predictive approaches that analyse larger datasets while retaining interpretability. The TexCoMP framework offers a practical, interpretable, and validated tool for evidence-based tribological engineering, supporting global safety standards and helping reduce slip-related incidents in built environments.

## Supporting information

S1 Data Minimal dataset. Minimal dataset underlying the findings reported in this article. Contains: – Aggregated mean and standard deviation of dynamic coefficient of friction (DFC) measurements for all experimental combinations (Sheet: DFC_Means_SDs) – Individual replicate-level DFC measurements with direction (Vertical/Horizontal) for full transparency and reproducibility (Sheet: DFC_Raw_Replicates) – Experimental metadata, instruments, conditions, and notes (Sheet: Metadata) These data support all statistical analyses, figures, and TexCoMP model validation.
(XLSX)

## Abbreviations

ACT: After Coating Treatment
ADA: Americans with Disabilities Act
ANSI: American National Standards Institute
ASTM: ASTM International (formerly American Society for Testing and Materials)
BCT: Before Coating Treatment
CDC: Centers for Disease Control and Prevention
CMP: Coating Material Performance
CM 1: Acrylic-based Coating Material No. 1
CM 2: Epoxy-based Coating Material No. 2
CM 3: Acrylic polymer-based Coating Material No. 3
CM 4: Acid-based Etchant Coating Material No. 4
COF: Coefficient of Friction
CT 1: Ceramic Tile No. 1
CT 2: Ceramic Tile No. 2
CT 3: Ceramic Tile No. 3
CT 4: Ceramic Tile No. 4
DFC: Dynamic Coefficient of Friction
E: Environmental
ISO: International Organization for Standardization
LOOCV: Leave-One-Out Cross-Validation
MAE: Mean Absolute Error
ML: Machine Learning
NFSI: National Floor Safety Institute
NR: Nitrile Rubber
OSHA: Occupational Safety and Health Administration
***R***_***a***_: Average Roughness
***R***_***q***_: Root Mean Square Roughness
***R***_***p***_: Maximum Peak Height
***R***_***t***_: Maximum Peak-to-Valley Height
***R***_***v***_: Maximum Depth
***R***_***z***_: Mean Roughness Depth
PCA: Principal Component Analysis
RMSE: Root Mean Square Error
RSS: Residual Sum of Squares
SH 1: Shoe No. 1 (Nitrile Rubber)
SH 2: Shoe No. 2 (Nitrile Rubber)
SH 3: Shoe No. 3 (Urethane Rubber)
STM: Surface Texture Modification
TexCoMP: Texture-Coating Material Performance
UR: Urethane Rubber
VIF: Variance Inflation Factors
WC: Without Coating Treatment
